# Clinical and Cost-Effectiveness of Blended Cognitive Behavioral Therapy or Psychodynamic Therapy Versus Face-to-Face Psychotherapy for Depression (BLENDED Study): Protocol for a Pragmatic, Multicenter, Assessor-Blinded Randomized Controlled Noninferiority Trial

**DOI:** 10.2196/80511

**Published:** 2026-01-14

**Authors:** Patrick Luyten, Danielle Speybrouck, Peter Martin, Stephan Claes, Nick Midgley, Hester van Eeren, Jan van Busschbach, Eileen Tang

**Affiliations:** 1 Research Department of Clinical, Educational and Health Psychology, University College London, London, United Kingdom; 2 Faculty of Psychology and Educational Sciences, KU Leuven, Tiensestraat 102 box 3722, Leuven, 3000, Belgium, 32 16-32-61-35; 3 Institute of Epidemiology and Health Care, University College London, London, UK; 4 University Psychiatric Center KU Leuven, Universitair Ziekenhuis Leuven, Leuven, Belgium; 5 Erasmus University Rotterdam, Rotterdam, The Netherlands

**Keywords:** depression, cognitive behavioral therapy, psychodynamic therapy, blended, cost-effectiveness

## Abstract

**Background:**

Depression is a highly prevalent disorder. Yet, there is still a considerable treatment gap because of capacity issues across clinical services, which create barriers to access to effective psychological therapies. In addition, many individuals with depression do not seek treatment, and waiting lists for psychotherapy are typically very long. Blended psychotherapy, which combines online components and in-person sessions, may help bridge the treatment gap as a cost-effective intervention that complements other types of treatment for depression, as it may reduce therapist time and potentially lower the threshold for people to seek treatment for their depression.

**Objective:**

This study aims to investigate the clinical effectiveness and cost-effectiveness of blended psychodynamic therapy (PDT) and cognitive behavioral therapy (CBT) for depression compared with face-to-face (FTF) PDT and CBT.

**Methods:**

A pragmatic, single-blind, multisite, noninferiority trial will randomize adult patients referred to mental health care centers in Flanders, Belgium, and diagnosed with major depressive disorder (MDD; n=504), to FTF or blended PDT and CBT. The primary outcome is to investigate whether blended therapy for depression (ie, blended PDT and CBT) is noninferior from baseline to 6-month follow-up after treatment termination compared with FTF PDT and CBT in terms of severity of depression assessed with the Beck Depression Inventory-II (BDI-II) based on intention-to-treat analyses. Secondary outcomes include severity of depression as measured with the BDI-II at 1 and 2 years after treatment termination; recovery from depression as assessed with the Structured Clinical Interview for DSM-5 disorders – Clinical Trials Version (SCID-5-CT) and the Patient Health Questionnaire-9 (PHQ-9) at treatment termination and at 6-month, 1-year, and 2-year follow-up; and quality of life as measured with the EuroQoL 5-Dimension 5-Level (EQ-5D-5L) at treatment termination and at 6-month, 1-year, and 2-year follow-up. The feasibility of implementing blended care will be investigated, and health economic analyses will address the cost-effectiveness of blended care versus FTF psychotherapy. Exploratory analyses will focus on possible predictors of treatment outcome and mechanisms of change. Sensitivity analyses will address the potential impact of the COVID-19 pandemic on therapeutic outcomes. Finally, a qualitative substudy aims to address patients’ and therapists’ subjective experience of blended psychotherapy.

**Results:**

The study was funded in July 2018, and the first patient was included in April 2019. As of September 2025, we have enrolled 463 patients. The first data lock (primary outcome) will take place in October 2025, and the results of the primary outcome are expected in February 2026. The second data lock is expected in March 2027, and the results of the 2-year follow-up are expected in September 2027.

**Conclusions:**

This trial promises to inform decisions concerning the implementation of blended versus FTF therapy for individuals with depression in routine clinical care.

## Introduction

### Background

Depression is a highly prevalent and disabling disorder that is associated with high psychosocial and economic costs. Population-based studies suggest a lifetime prevalence of approximately 15% for unipolar depression and up to 25% in women [1,2], with some evidence suggesting increases in prevalence in recent decades [3], particularly in young people [4-6]. However, there is still a considerable treatment gap because of capacity issues across clinical services, which create barriers to access to effective psychological therapies [7-10]. In addition, studies show that many people with depression do not seek treatment or delay seeking treatment [8,11,12].

There is, therefore, an urgent need to develop and implement (cost-)effective treatments for depression in routine clinical practice and decrease the barriers to treatment. Internet-based therapy, particularly when offered in a blended format, in which online work is integrated into and alternated with in-person face-to-face (FTF) sessions, may help to bridge this treatment gap as a potentially cost-effective treatment that complements other treatments for depression [7,9,10,13-16]. Blended psychotherapy has the potential to lower the threshold for people with depression to seek help, as studies have suggested that people feel that they are more in charge of their own therapeutic process with blended treatment compared with traditional FTF therapy [15,17]. Moreover, blended treatment for depression typically reduces therapist time and thus increases capacity [9,18,19]. Hence, the implementation of blended treatments for depression in routine clinical care may lead to increased availability of psychotherapy for depression and a more effective use of available resources in mental health care.

Although a number of recent high-quality studies and meta-analyses have suggested that blended psychotherapy may be as effective as traditional FTF psychotherapy for depression [9,20-30], there is a need for further research, particularly concerning the (cost-)effectiveness of blended psychotherapy for depression in patients with more complex presentations [17,19,22,31-37]. Moreover, little is known about the effects of blended psychotherapy for depression in the long term [21,22,31,33]. Furthermore, most research in this area has investigated the effectiveness of blended cognitive behavioral therapy (CBT), although there is also growing evidence for the effectiveness of other types of internet-delivered treatments, including psychodynamic therapy (PDT) [38]. Most studies of internet-delivered PDT have focused on adolescents with depression and involved guided internet-delivered PDT, which contained lower levels of therapist guidance than in blended treatment [39-41]. Finally, it is largely unknown which factors may moderate or mediate therapeutic change in blended treatment for depression [15,27,30], and studies have suggested that although therapists and patients both report advantages associated with blended psychotherapy, they also experience disadvantages and barriers in relation to blended psychotherapy for depression in terms of acceptability and suitability [28,42-44].

This study, therefore, set out to investigate the (cost-)effectiveness of blended PDT and CBT for depression compared with FTF PDT and CBT in adults diagnosed with major depressive disorder (MDD) from baseline to 12-month follow-up, in a pragmatic, single-blind, multisite, noninferiority trial in secondary mental health care centers in Belgium. Given the recurring nature of depression and the relatively high relapse rates following brief treatments for depression [45,46], patients will also be followed up until 2 years after the end of treatment. A health economic evaluation will address the potential cost-effectiveness of blended psychotherapy versus FTF psychotherapy for depression. Exploratory analyses will focus on potential moderators and mediators of change. A qualitative substudy will investigate both patients’ and therapists’ experiences of blended treatment, with a focus on acceptability, credibility, and satisfaction with treatment.

In what follows, we discuss the emerging evidence for the effectiveness of internet-based psychotherapy, with a focus on blended treatments. We then provide an outline of this study, methods, and expected results, reported according to the SPIRIT (Standard Protocol Items: Recommendations for Interventional Trials) guidelines [47].

### Internet-Based Psychotherapy and the Need to Improve Access to Psychological Therapies

Meta-analyses and qualitative reviews suggest that internet-based psychotherapy may be as effective as FTF psychotherapy for MDD both at treatment termination and at follow-up in the medium to long term [15,21-23,27,33,35,37]. Research in this domain to date has mainly focused on 2 types of internet-based psychotherapy, CBT [15,48] and PDT [38,49]. Although few trials have directly compared these 2 types of internet-based therapy, existing studies suggest that they have similar effects [38,40]. These findings are also consistent with findings on FTF psychotherapy, with meta-analyses showing no substantial differences in the effectiveness of brief CBT and PDT for MDD [50-54].

Research findings have also suggested that guided internet-delivered treatments may be associated with better outcomes and lower dropout rates compared with unguided or minimally guided online treatments [17,22,28,55,56]. These findings have sparked increasing interest in blended psychotherapy; this type of internet-delivered treatment may combine the “best of both worlds” in that it involves alternating FTF therapy sessions and online modules. In this format, therapists are able to attend to so-called process-related components of psychotherapy (ie, building a therapeutic alliance, explaining the treatment approach, and addressing specific needs, concerns, and anxieties), which may increase adherence to treatment and reduce dropout [10,28,43,57]. More content-related components of the treatment can be the focus of the online modules, which may be flexibly integrated among the FTF sessions. Moreover, personal contact with a therapist may broaden the spectrum of patients who can be treated with blended psychotherapy compared with unguided or minimally guided internet-delivered treatments [17,58]. Blended psychotherapy may also foster feelings of autonomy and empower patients to a greater extent than FTF therapy, as the availability of online modules may better enable patients to work on their presenting problems between FTF sessions, which may further facilitate the translation of insights from therapy to their everyday lives [59]. Finally, blended psychotherapy may also be more cost-effective than FTF psychotherapy, as it typically involves less therapist time compared with FTF therapy [9,18,19,60].

More research is needed concerning the (cost-)effectiveness, acceptability, and suitability of blended treatment for depression for a number of reasons. First, the number of studies comparing the effectiveness of blended psychotherapy for depression with FTF psychotherapy is relatively small [21,22,33], and even fewer studies have compared their cost-effectiveness [60]. For instance, a recent meta-analysis [22] included only 9 randomized controlled trials (RCTs) of blended psychotherapy for depression versus control conditions, with considerable heterogeneity among studies in terms of the kind of blended treatments offered (with digital sessions being either integrated or added as supplementary treatment), the ratio of FTF sessions to online modules, and the number of FTF sessions and online modules. Second, the majority of studies of blended psychotherapy for depression have been conducted in controlled research settings, and thus it remains uncertain to what extent these findings can be generalized to more complex patients typically seen in routine clinical care [35,58]. Hence, there is a need for more research on the suitability of internet-based treatments for different types of patients with depression and to identify factors that foster or hinder therapeutic change in these types of treatments [15,37,40,44,61,62]. In this context, it is noteworthy that there is still very little research on predictors of outcome or mechanisms of change in blended versus FTF psychotherapy for depression, although research on these issues could have important implications for assessing the suitability of blended versus FTF psychotherapy for different patients.

Finally, there is an emerging body of evidence from qualitative studies focusing on the subjective experience of both patients and therapists involved in blended care for depression, but more research is needed in this context as well. Studies on internet-based interventions in general tend to highlight the potential importance of human interaction and support in explaining treatment outcomes in blended treatments across different therapeutic modalities [63-65], whereas a lack of understanding, trust, and support has been associated with dropout and lack of improvement [66-71]. Whether this is also the case for blended treatment for depression is largely unknown, given the small number of studies. Moreover, almost all of these studies have examined internet-based CBT [15,38]. Only a few qualitative studies have investigated the role of therapists and the therapeutic relationship in other treatment modalities, such as PDT, and most of these studies have focused on adolescents with depression [62,72,73].

### This Study

The primary objective of this study is to investigate whether blended therapy for depression (ie, blended PDT and CBT) is noninferior from baseline to 6-month follow-up after treatment termination compared with FTF PDT and CBT in adults diagnosed with MDD (target n=504). FTF psychotherapy will consist of 16 sessions of PDT or CBT, offered over a period of 6 months. Blended therapy will consist of, on average, 8 FTF sessions, alternating with 8 online modules that patients can complete between FTF sessions, similarly offered over a period of 6 months to control for time effects. The primary outcome assessment will focus on noninferiority in terms of severity of depression assessed with the Beck Depression Inventory-II (BDI-II) [74] from baseline to 6 months after the end of treatment (ie, 12 months after the start of treatment), based on intention-to-treat (ITT) analyses. The noninferiority margin was defined as a difference in effect size (ES) equal to or smaller than 2 points on the BDI-II between blended care and FTF psychotherapy from baseline to 6-month follow-up after treatment termination. Given an estimated baseline SD of 10, this is equivalent to a standardized ES of Cohen *d*≤0.2, which is below the minimal meaningful difference in psychosocial interventions for depression proposed as Cohen *d*=0.24 [75].

The secondary objectives of this study are 6-fold. First, the trial aims to investigate the comparative efficacy of blended psychotherapy versus FTF psychotherapy on the following secondary outcomes: severity of depression as measured with the BDI-II at 1 and 2 years after treatment termination; recovery from depression as assessed with the Structured Clinical Interview for DSM-5 disorders – Clinical Trials Version (SCID-5-CT) [76] and the Patient Health Questionnaire-9 (PHQ-9) [77] at treatment termination and at 6-month, 1-year, and 2-year follow-up; and quality of life as measured with the EuroQoL 5-Dimension 5-Level (EQ-5D-5L) [78] at treatment termination and at 6-month, 1-year, and 2-year follow-up.

Second, we aim to investigate the feasibility of implementing blended care in the participating mental health care centers in Belgium, including the acceptability of treatments to both therapists and patients. To this end, we will record the recruitment rate, retention in treatment, treatment adherence, and adherence to the research protocol. Acceptability will be indicated by the number of sessions attended, including the number of individuals who refuse treatment, and feasibility will be indicated by the number of patients who do not comply with the full clinical and research protocol. Patients will also complete measures of credibility of the treatments and satisfaction with the treatments. To measure patient attitudes toward the treatment programs, we will administer the Credibility and Expectancy Questionnaire (CEQ) [79] at baseline and an adapted version of this measure at posttreatment and 6-month follow-up. Satisfaction with treatment will be measured with the Client Satisfaction Questionnaire-8 (CSQ-8) [80]. Treatment integrity of therapists will be assessed on the basis of independent ratings of audio- or video-recorded psychotherapy sessions.

Third, the cost-effectiveness of blended care versus FTF psychotherapy will be investigated based on a societal perspective, taking all relevant costs and effects into account (intervention costs, direct and indirect medical costs, as well as productivity losses and costs incurred elsewhere in the health care system) based on both self-reported data, using an adapted version of the Treatment Inventory of Costs in Patients with psychiatric disorders (TIC-P) [81], and data provided by the Rijksinstituut voor Ziekte- en Invaliditeitsverzekering/Institut National d’Assurance Maladie-Invalidité (RIZIV/INAMI), the Belgian federal national health insurance body.

Fourth, exploratory analyses will focus on possible predictors of treatment outcome and mechanisms of change. Consistent with the focus of this study on patients who are typically referred to specialized mental health care centers, these analyses will focus on patient features that have often been linked to negative outcomes in the treatment of depression [57,82,83], such as severity of depression (assessed with the BDI-II and PHQ-9), dependent and self-critical personality features (assessed with a brief version of the Depressive Experiences Questionnaire [DEQ]) [84], and patients’ experience of both types of treatment as assessed with the CEQ [79]. Concerning mechanisms of change, relationships between changes in dependent and self-critical personality features assessed with the DEQ [84] and primary and secondary outcomes will be investigated from both a variable-centered and a person-centered perspective.

Fifth, sensitivity analyses will address the potential impact of the COVID-19 pandemic on therapeutic outcomes.

Sixth, a qualitative substudy aims to address patients’ and therapists’ subjective experience of blended psychotherapy, with a focus on treatment suitability and the advantages and disadvantages of blended therapy. This qualitative study aims to capture the subjective experiences and understandings of both patients and therapists, including what they may find helpful or hindering in blended psychotherapy. The study thus also includes a strong service-user perspective, which is often neglected in psychotherapy outcome research. Qualitative research regarding the subjective experience of blended psychotherapy for depression may also provide policymakers and clinicians with detailed and in-depth information about how to contextualize, and thus better understand, findings from RCTs, which will help them effectively adapt and implement blended psychotherapy in routine clinical care in the future [85].

## Methods

### Trial Design

This study is a pragmatic, single-blind, multicenter, randomized controlled, noninferiority, multiple-armed trial, with patients being followed up for up to 2 years after treatment termination. The primary interest lies in comparing the efficacy of blended psychotherapy versus FTF psychotherapy for depression at 6-month follow-up after treatment termination. The overall expectation is that blended psychotherapy is noninferior to FTF psychotherapy but more cost-effective. The noninferiority margin is defined as a small difference in ES between blended and FTF psychotherapy (2 points on the BDI-II), which is deemed clinically meaningful in psychotherapy research [75,86]. Additionally, the study design allows exploration of the relative effectiveness of the 2 different types of psychotherapy (ie, PDT vs CBT) in both forms provided (blended and FTF), although the trial is not powered for this purpose, and this is not the main aim of the study. A qualitative substudy will be embedded within the trial, focusing on the subjective experiences of blended therapy of both therapists and patients. Semistructured interviews, based on an adapted version of the Experience of Therapy Interview [87], will be conducted with 16 patients who found blended psychotherapy to be either helpful or unhelpful. Focus groups will be conducted with participating therapists to explore their experiences of delivering blended psychotherapy. Qualitative data will be analyzed using thematic analysis (TA) based on a combined deductive–inductive approach.

### Study Setting

The study will be conducted in specialized, state-funded secondary care mental health care centers (Centra Geestelijke Gezondheidszorg [CGGs]) in Flanders, Belgium. About half of patient referrals to CGGs are made by primary care medical practitioners or specialized mental health professionals, about one-third are self-referrals, and the remaining patients are referred by schools, family, or friends, or by the Department of Justice. We anticipate that a maximum of 15 CGGs will participate in the study. Factors that might hamper accrual are expected to be mainly related to the severity and complexity of the presenting problems of patients at CGGs, with a substantial number of patients needing longer-term treatment and/or counseling. Access to the internet might be a challenge for some depressed patients seen at these centers. However, 99.9% of people in Belgium potentially have access to broadband internet (>30 Mbps). In 2015, when this study was planned, 81.8% of households had access to the internet, and most of those had access to broadband internet. Access to the internet was slightly less prevalent among people living in poverty in Belgium. Patients seeking treatment for depression overall have higher levels of poverty than the general population. This is especially the case for patients referred to CGGs, with an estimated 40%‐45% of these patients meeting criteria for poverty (defined as having low levels of education and being unemployed). However, data from 2015 showed that, of people living in poverty in Belgium, 72% had internet access, and this proportion has increased year on year. For the majority of patients in this study, therefore, internet access is not expected to differ greatly from that of the general population (ie, minimum 72% for patients vs 81.8% in the general population, based on 2015 data), and thus it is likely to have a relatively limited impact on inclusion rates [88].

### Participants

In total, 504 patients with MDD will be included. Based on a review of referrals over the 3 years preceding the trial in the participating CGGs, we anticipate a recruitment rate of approximately 168 patients with MDD who can be included per 6 months (ie, approximately 28 patients per month, approximately 34 patients per CGG in the 18-month recruitment period, and approximately 2 patients per month per CGG).

### Eligibility Criteria

Inclusion and exclusion criteria for the trial are depicted in Textbox 1. Patients excluded from the trial will receive standard care in one of the participating centers or will be referred to specialist care, as appropriate.

Textbox 1. **Inclusion and exclusion criteria.**

Inclusion criteria:

- Aged 18-65 years.
- Current diagnosis of major depressive disorder (MDD) with or without dysthymic disorder according to *Diagnostic and Statistical Manual of Mental Disorders, Fifth Edition* (*DSM-5*) criteria [89].
- Patient Health Questionnaire-9 (PHQ-9) score ≥10.
- The use of pharmacotherapy for depression and other psychiatric disorders during the intervention is allowed (with the exception of antipsychotic medication for psychotic disorder; see exclusion criteria), as this is a pragmatic trial and many patients seen in routine clinical care use pharmacotherapy.

Exclusion criteria:

- Current psychotic symptoms or bipolar disorder.
- Current use of antipsychotic medication specifically for the treatment of primary psychotic disorder.
- Severe personality disorder (eg, borderline personality disorder).
- Historic or current self-injury/parasuicide of such an extent and/or severity that it may substantially interfere with the ability to engage in brief psychotherapy.
- Current excessive use of drugs/alcohol.
- Lack of Dutch language fluency.
- Clinical contraindication to brief psychotherapy (eg, attachment history characterized by multiple separations, serious ongoing trauma in childhood, or multiple caregivers, suggesting the need for longer-term psychotherapy).
- Evidence of pervasive use of help.
- Highly unstable or insecure life arrangements.
- No access to a computer/internet or computer illiteracy.
- Participation in another depression clinical trial within the past year in which the participant had received CBT or PDT.

For the qualitative study, patients will be recruited based on purposive sampling to identify participants who found blended therapy either helpful or unhelpful. After completing the 6-month follow-up assessment, patients from the blended psychotherapy conditions will be asked to rate the level of helpfulness of blended psychotherapy on a scale from 0 (Not at all helpful) to 100 (Extremely helpful). They will mark their score on a form that is sent to them by email, after completion of a separate informed consent procedure specific to the qualitative study. Patients with scores either below 35 or 65 or higher will be eligible to participate in the qualitative study and will be categorized as those who found blended therapy to be “not helpful” or “helpful,” respectively. We will thus recruit an equal number of participants from each group from both blended CBT and blended PDT, to give a total of 16 patients (4 “helpful” and 4 “not helpful” cases each from blended CBT and blended PDT). Therapists for the focus groups will be contacted via email and/or telephone. All therapists who have participated in the trial will be eligible for the qualitative substudy.

### Recruitment, Screening, and Gaining Informed Consent

Each participating mental health care center will be responsible for identifying potential patients who meet eligibility criteria for the study. Identification of potential participants will occur at the point of triage or initial assessment, depending on the specific procedures of the participating centers, as part of routine clinical care. Specifically, the clinician conducting initial assessments of patients referred for mood problems will consider the inclusion and exclusion criteria for the study. It is acknowledged that the assessing clinician may identify exclusion criteria or other factors that could deem a patient unsuitable for the study (eg, risk of possible injury to therapists, imminent suicidal risk) even if the patient meets the inclusion criteria. Unless this is the case, the clinician will offer the patient a brief verbal introduction to the study, constituting a preliminary invitation to participate. The clinician will provide the patient with a participant information sheet to read and keep. The participant information sheet will include age- and culturally appropriate material explaining the study and what involvement in the study would entail at that particular site. Clinicians then confirm whether the patient consents to being contacted by a researcher from the research team, who will then discuss the study in more detail with the patient. Patients will be given ample time (minimum 48 hours) to think about the information provided and can request more time to think about the study before consenting to further contact from the researcher. If a patient verbally consents to being contacted by a research assistant (RA), the clinician informs the research team of this decision and provides relevant contact information.

An RA will contact potential participants no less than 48 hours after they receive information about the study from participating centers, to explain what participation might involve and to arrange an appointment at the mental health center. A study identification number will be assigned to the participant, and from this point on, the participant will be referred to only by this number, and their collected data will be labeled with it. After the participant has provided explicit and written informed consent, an RA will administer the SCID-5-CT interview and the PHQ-9 and will assess all other inclusion/exclusion criteria. When all the measures have been completed, and study eligibility has been confirmed at baseline (ie, checks for inclusion/exclusion criteria have been confirmed), an RA will provide the trial coordinator (TC) with the details required to randomize the participant to one of the four conditions.

For the qualitative substudy, an RA will invite (first via email and then followed up by telephone) all patients who received blended treatment in the BLENDED trial who are beyond the 6-month follow-up assessment to find out whether they are interested in participating in an interview study focusing on their experience of blended psychotherapy. A minimum of 48 hours will be allowed between the initial contact with the participant and confirmation of their participation (by telephone or email) and the participant being approached again by the RA. An information leaflet and an additional informed consent form (ICF) will be sent by email to the patient following this first contact. This schedule is to allow sufficient time for individuals to consider and discuss participation in the substudy with any party they may choose to consult. An RA will confirm with the potential participant that this time frame has been sufficient and allow additional time if requested. As the substudy will be conducted online and not at the mental health care centers, patients will be asked in a separate contact to sign and email the ICF to the research team; after receipt, the RA will sign the ICF and return the fully signed ICF to the patient. As mentioned earlier, eligibility (ie, whether they found blended psychotherapy helpful or not) will be determined based on patients’ self-reported levels of helpfulness of blended treatment when they are approached to participate in this study. If a patient is eligible for the qualitative study, an RA will contact the patient again to schedule an online interview. For the focus group study with therapists, a similar procedure will be followed to obtain informed consent from the therapists.

### Randomization

This trial will use stratified and automated randomization of participants to one of four conditions in permuted blocks built into the electronic Case Report Form (eCRF). Stratification will be carried out in 2 stages. At stage 1, patients will be randomized to therapists within each participating center, using permuted blocks to ensure that the maximum difference of trial caseload for therapists within the same center is on average 2 patients. As each therapist will provide only either CBT or PDT, stage 1 implicitly allocates patients to the type of therapy. At stage 2, patients will be randomized to either FTF or blended care, again using permuted blocks such that the maximum difference in the number of FTF and blended therapy patients for each therapist will be 2 patients at any one point in time. Participant randomization will be initiated centrally by the TC, following the completion of the eligibility assessment (screening) by the RA and of the baseline measures by the participant, and before commencement of any trial intervention. As randomization will be performed using an automated web-based randomization algorithm built into the eCRF, which sends an automated email to the therapist, this will ensure that the RA remains blind to the treatment condition to which the patient is randomized.

This 2-stage stratified randomization scheme ensures balance for the comparison of FTF and blended therapy with respect to participating centers and therapists. The scheme will also promote, although not guarantee, balance with respect to any patient-level prognostic factors whose distribution varies between the patient populations of the centers (eg, age).

### Interventions

Both CBT and PDT, as offered in this trial, are based on manualized treatments developed in the context of the Improving Access to Psychological Therapies program in the United Kingdom [90,91]. FTF PDT and CBT consist of 16 FTF sessions, offered over a period of 4‐6 months. Blended PDT and CBT consist of 8 FTF sessions in combination with 8 online modules offered over a period of 4‐6 months and thus are similar in length to the FTF psychotherapies. Blended PDT and CBT are based on principles outlined in the manualized FTF psychotherapies and adapted for use in Flanders (Belgium) [17,92] in collaboration with service users and therapists as part of depressiehulp.be, a government-supported online platform that offers psychoeducation and (both unguided and guided) internet-delivered treatment for depression.

FTF sessions in FTF therapy and blended therapy are offered on a weekly and a 2-weekly basis, respectively, although with mutual agreement by the patient and therapist, the session frequency may deviate from the planned weekly or 2-weekly sessions. 

The content of FTF psychotherapy and blended therapy is very similar, as the treatments follow the same treatment manuals and treatment phases. The main difference between FTF and blended therapy is that in blended therapy, the therapists will be specifically trained to help motivate and encourage patients to complete the online modules between the FTF sessions, and to take the patient’s experience in completing each module as the starting point of the next FTF session. Hence, using the terminology introduced by Erbe et al [23], in this study, blended therapy will be offered in a fully integrated format, with digital modules being a core component of the treatment, FTF and digital sessions alternating, and blended treatment primarily being delivered in a standardized format for each patient, with some room for personalization of the treatment as detailed below.

FTF and blended PDT will be based on dynamic interpersonal therapy (DIT), an integrative evidence-based brief treatment for depression [90,93]. FTF DIT involves 16 weekly sessions of individual therapy, which formulates the presenting symptoms of depression as responses to interpersonal difficulties or perceived threats to attachments (loss and/or separation) and hence also as threats to the self. DIT conceptualizes depression in terms of an underlying temporary disorganization of the attachment system caused by current relationship problems, which in turn generates a range of distortions in thinking and feelings typical of depression. In DIT, there is a focus on both content and process. The content focus relates to the content of the individual dynamics that are associated with the onset and maintenance of depression. This focus helps the patient understand the connection between their presenting symptoms and what is happening in their relationships by identifying a core, repetitive pattern of relating that becomes the focus of the therapy, which is known as the interpersonal affective focus (IPAF). The process focus involves fostering the patient’s capacity for reflective functioning or mentalizing, as depression is typically associated with serious impairments in this capacity. In DIT, therapists use a range of interventions and techniques to restore the capacity for reflective functioning and to foster insight and change in the IPAF, consisting of supportive, mentalizing, insight-oriented, and directive interventions to empower the patient to translate insights obtained in therapy to their everyday life.

DIT consists of 3 phases. In the first phase (sessions 1‐3), the focus is on engaging and motivating the patient, assessing their presenting problems and dynamics, and collaboratively delineating the focus of the treatment (ie, the IPAF). The middle phase (sessions 4‐12) focuses on exploring how the patient’s typical pattern of relating to the self and others has led to feelings of depression, and how a change in this pattern might improve their mood. The ending phase (sessions 13‐16) is mainly concerned with empowering the patient to translate the insights they have gained in therapy to their everyday life.

The blended DIT program consists of the same 3 phases as FTF DIT: an exploration phase, a middle or working-through phase, and an ending phase. Instead of having 16 FTF sessions, patients in blended DIT are invited to complete online modules between 8 FTF sessions to explore the typical dynamics that are otherwise explored in the FTF sessions, and to discuss these in subsequent FTF sessions. The structure and phases of blended DIT are discussed in detail elsewhere [92].

FTF CBT involves 16 sessions offered over 4‐6 months. CBT is based on the assumption that depression is directly related to patterns of thinking [91,94]. Specifically, dysfunctional and often automatic patterns of thinking are assumed to be related to the onset and maintenance of depression. The overall goal of CBT is therefore to help a person recognize such patterns of thought, evaluate their validity, and replace them with more adaptive ways of thinking. In CBT, there is an emphasis not only on thinking (ie, “cognitive”), but also on doing and thus on changing behavior (“behavioral”).

Therapeutic sessions in FTF CBT are highly structured, with clear tasks and goals in each session. These tasks and goals draw from more classic cognitive behavioral principles, including psychoeducation about depression, cognitive restructuring (ie, identifying and challenging maladaptive thoughts and beliefs, fostering problem-solving capacities), behavioral activation, and relapse prevention. Therapists are allowed to use these principles flexibly in a manner that is tailored to the needs and preferences of individual patients. In this trial, a focus on more recent principles and techniques that have emerged within the CBT tradition, such as fostering mindfulness and applying principles of acceptance and commitment therapy, is also part of both FTF and blended CBT [91], but these are covered in only one FTF session or one online module.

The structure and focus of FTF CBT and blended CBT are similar. The only difference is that, as with FTF and blended PDT, in blended CBT, 8 of the sessions are replaced by online treatment modules, so that the focus in the FTF sessions is more on attending to the process components of treatment (ie, is the patient motivated? Do they understand the treatment rationale?) and on reviewing and discussing therapeutic progress. The structure and phases of blended CBT in this trial are discussed in detail elsewhere [17].

As a result of the early phase of the COVID-19 pandemic, various restrictions made physical attendance of screening and therapeutic sessions impossible. An amendment to the original trial protocol, replacing FTF in-person sessions with online sessions, was allowed during this phase of the pandemic and particularly during mandated restrictions on meeting in person (so-called “lockdowns;” see protocol amendments in Multimedia Appendix 1). Sensitivity analyses will address the potential effects on treatment outcomes of the fact that some patients had their FTF sessions online (see also the Statistical Analysis Plan section).

As this is a pragmatic trial, if the patient and therapist agree at the end of the treatment program that further treatment is necessary, the treatment can be continued with the same therapist, or the patient can be referred for additional and/or longer-term therapy based on mutual agreement between patient and therapist. In the former case, the number of additional sessions with the same therapist is recorded in the eCRF; in the latter case, additional psychotherapy is monitored using the TIC-P.

Therapists will be certified CBT and PDT psychotherapists (psychiatrists or psychologists), who must have completed a postgraduate psychotherapy training of 3‐4 years. In addition, they will receive a 4-day training program in manualized FTF and blended CBT or PDT delivered by certified trainers in CBT and PDT, respectively. They will also receive 2-weekly small-group supervision of at least 2 patients by certified CBT and PDT supervisors.

### Discontinuation and Withdrawal

Participants can withdraw from the study for any reason at any given time. Therapists can propose to withdraw a patient from the study (1) if they are of the opinion that a patient risks serious deterioration and nonresponse to the treatment to which they have been allocated (ie, the patient needs longer-term treatment), and/or (2) if it becomes clear that the patient does not meet inclusion criteria or meets one or more exclusion criteria, despite initial screening and assessment (eg, the patient does have severe personality disorder), and/or (3) if participation of the patient in the study seriously interferes with treatment (eg, the patient is overly burdened by having to complete assessments). In all of these cases, the therapist needs to discuss the option of withdrawal from the study with the patient and must seek advice from a site supervisor and from the chief investigator. Patients who withdraw from the study for any reason are not replaced.

### Measures

**Figure 1.**
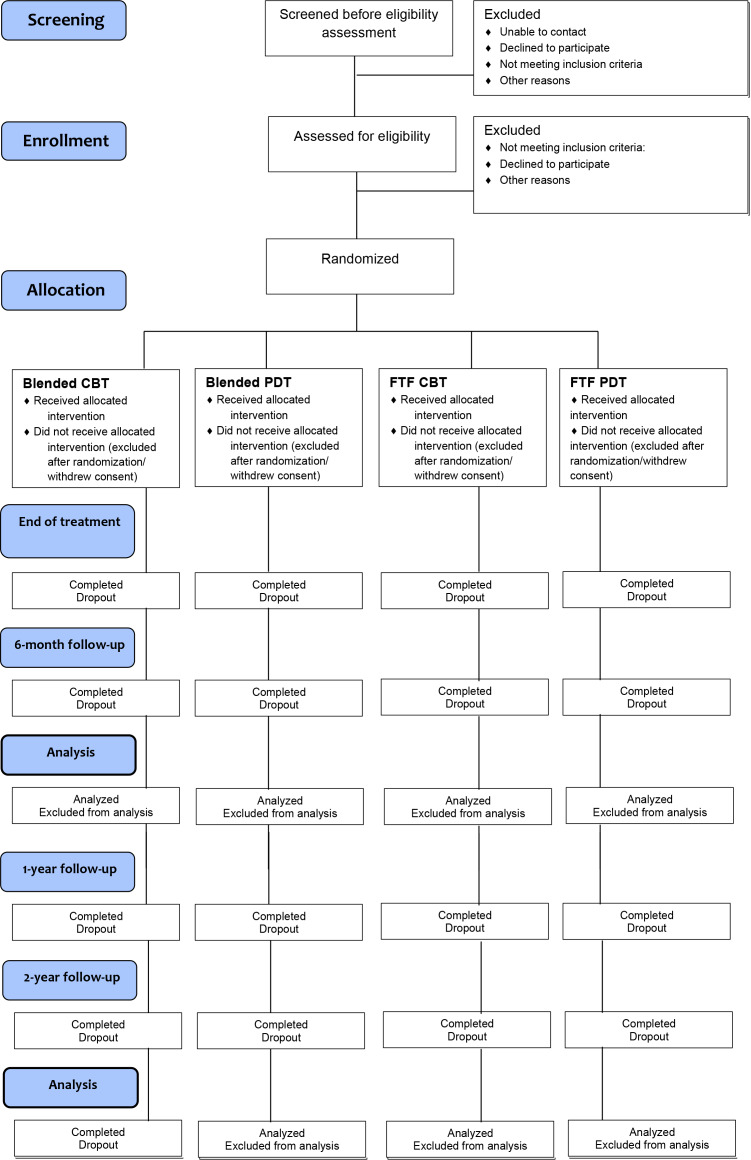
CONSORT (Consolidated Standards of Reporting Trials) diagram of the BLENDED trial. CBT: cognitive behavioral therapy; PDT: psychodynamic therapy; FTF: face-to-face.

The following measures will be completed by patients during the trial as specified in the CONSORT diagram (Figure 1; also see Table 1). Visit windows are 2 months either side of the ideal follow-up time point.

The primary outcome will be the severity of depression as measured by the BDI-II [74] assessed at 6-month follow-up after treatment termination. The BDI-II consists of 21 items, in line with the depression criteria of the *Diagnostic and Statistical Manual of Mental Disorders, Fifth Edition* (*DSM-5*) [89]. The BDI-II will also be administered at 1-year and 2-year follow-up for secondary outcome analyses.

Secondary outcomes include:

- Recovery (defined as no longer scoring above the clinical cut-off for complete or partial recovery), assessed at treatment termination and at 6-month, 1-year, and 2-year follow-up by using the SCID-5-CT [76]. The SCID-5-CT is customized to include only those diagnostic elements that are needed to determine whether the patient fulfills the inclusion and exclusion criteria of a particular clinical trial. Recovery will also be assessed with the PHQ-9, a brief, 9-item multipurpose instrument for screening, diagnosing, and monitoring the severity of depression [77]. It incorporates the *DSM-5* diagnostic criteria for depression and is extensively used in research on depression and in routine clinical practice.
- Quality of life as measured with the EQ-5D-5L [78] at baseline, treatment termination, and at 6-month, 1-year, and 2-year follow-up. The EQ-5D-5L contains 5 items, measuring quality of life in 5 dimensions of health (mobility, self-help, habitual activities, pain, and anxiety/depression), each with 5 levels reflecting “no problems,” “slight problems,” “moderate problems,” “severe problems,” and “extreme problems.” The EQ Visual Analog Scale records the respondent’s self-rated health on a 20 cm vertical visual analog scale with endpoints labeled “the best health you can imagine” and “the worst health you can imagine.” The EQ-5D-5L will also be used for the cost-effectiveness component of the study.

As indicators of the acceptability of the treatment, we will also measure treatment expectancy (at baseline), treatment credibility (at follow-up), and satisfaction with treatment:

- Patients’ beliefs about the credibility of treatment and their expectations of the effectiveness of treatment will be measured with the CEQ [79] at baseline, and with a modified version of the CEQ at treatment termination and 6-month follow-up. The CEQ has 6 questions, rated on a 9-point Likert-type scale (items 1‐3 and item 5) or a 0%‐100% scale (items 4 and 6).
- Satisfaction with treatment will be measured at baseline, treatment termination, and at 6-month, 1-year, and 2-year follow-up, using the CSQ-8 [80]. This questionnaire contains 8 questions that are scored on a 4-point scale.

**Table 1.** Overview of the BLENDED trial procedures based on SPIRIT (Standard Protocol Items: Recommendations for Interventional Trials).

	Study period						
	Enrollment	Allocation	Postallocation				Close-out
Timepoint	*–t_1_*	0	During treatment	End of treatment	Follow-up at 6 months	Follow-up at 1 year	Follow-up at 2 years
				6 months after start of treatment (±2 months)	12 months after start of treatment (±2 months)	18 months after start of treatment (±2 months)	30 months after start of treatment (±2 months)
Enrollment							
Eligibility screen: PHQ-9^a^; SCID-5-CT^b^	✓						
Informed consent	✓^c^						
Allocation		✓^d^					
Interventions							
FTF^e^ DIT^f^			✓				
Blended DIT			✓				
FTF CBT^g^			✓				
Blended CBT			✓				
Assessments							
Demographics		✓					
Concomitant medications		✓		✓	✓	✓	✓
BDI-II^h^		✓		✓	✓	✓	✓
SCID-5-CT				✓	✓	✓	✓
PHQ-9		✓		✓	✓	✓	✓
TIC-P^i^		✓		✓	✓	✓	✓
EQ-5D-5L^j^		✓		✓	✓	✓	✓
CEQ^k^		✓		✓	✓		
CSQ-8^l^		✓		✓	✓	✓	✓
Safety assessment		✓	✓	✓	✓		
Qualitative interview					✓^m^		

a PHQ-9: Patient Health Questionnaire-9.

b SCID-5-CT: Structured Clinical Interview for DSM-5 Disorders – Clinical Trials version.

c Before eligibility screen.

d After baseline assessments.

e FTF: face-to-face.

f DIT: dynamic interpersonal therapy.

g CBT: cognitive behavioral therapy.

h BDI-II: Beck Depression Inventory-II.

i TIC-P: Treatment Inventory of Costs in Patients with Psychiatric Disorders.

j EQ-5D-5L: EuroQoL 5-Dimension 5-Level.

k CEQ: Credibility and Expectancy Questionnaire.

l CSQ-8: Client Satisfaction Questionnaire-8.

m Purposeful sample.

As part of the health economic evaluation, patients will complete the TIC-P [81], which has been used by the authors in previous RCTs [95,96]. The TIC-P is a generic questionnaire designed for self-report by patients with a psychiatric disorder and consists of 2 parts. The first part assesses relevant contacts with health care (eg, contacts with psychotherapists, inpatient care, visits to the primary care medical practitioner, etc). The second part consists of the Short Form-Health and Labor Questionnaire (SF-HLQ), which is designed to collect data on productivity loss resulting from health problems. Additionally, 3 questions are included to measure productivity losses due to reduced efficiency during paid work, if appropriate (see the Health Economic Evaluation section for more details). In addition, direct health-related costs will be calculated based on reimbursement data from RIZIV/INAMI, the national health service body in Belgium. Specifically, after the completion of the study, the sponsor will request approval from the competent chamber of the Information Security Committee to have the relevant study data linked with IMA (InterMutualistisch Agentschap/Agence InterMutualiste) data by a trusted third party (TTP, eHealth platform) using the participant’s national number. As soon as the approval has been received, the sponsor will collaborate with the eHealth platform and IMA for the data linkage process. This data linkage is planned to obtain a dataset containing costs related to health care paid by the compulsory health insurance and to compare these costs with self-reported direct and indirect costs assessed by the TIC-P, to arrive at a more comprehensive assessment of the cost-effectiveness of the interventions investigated in this trial.

### Serious Adverse Events

Only the serious adverse events (SAEs) listed below, occurring from the time of the participant providing written informed consent until 90 days after cessation of the trial treatment, will be recorded in the eCRF, and the sponsor of the study will be notified within 24 hours of the research staff at the site becoming aware of the event.

For each SAE, the following information will be collected:

- Full details in medical terms and case description.
- Event duration (start and end dates, if applicable).
- Action taken.
- Outcome.
- Seriousness criteria.
- Causality (ie, relatedness to trial intervention/investigation), in the opinion of the research team.
- Severity (mild, moderate, or severe).
- Whether the event would be considered expected or unexpected.

Any change of condition or other follow-up will be recorded, and the sponsor will be notified of such changes as soon as the information is available, preferably within 1 working day. Events will be followed up on until the event has resolved or a final outcome has been reached by recording on a detailed Safety Reporting Form. All SAEs will also be reported to the Trial Steering Committee (TSC). In the event that there are 5 or more SAEs within a period of 6 months, or any other condition that may give rise to serious concerns regarding the occurrence of SAEs (eg, when there is reason to suspect that the experimental condition may lead to more SAEs), an unblinded evaluation by an independent statistician and review by an independent psychiatrist or psychologist may be demanded by the TSC and reported back to the TSC. Depending on the outcome of this independent evaluation, the TSC can advise the research team to end the trial should there be sufficient concern about the welfare of patients in the trial.

The following SAEs will be monitored, recorded, and reported:

- Lethal suicide attempts and/or suicide attempts that require hospitalization.
- Any other behavior that may be related to depression that requires inpatient hospitalization or that may result in persistent or significant disability/incapacity (eg, serious self-harm, overdose).

### Blinding

In trials of psychosocial interventions, therapists and patients cannot be blinded to the type of intervention they provide and receive, respectively. To minimize the risk of potential biases in the assessment of outcomes arising from knowledge of treatment allocation, the main outcome data will be collected by an RA who will be unaware of each participant’s treatment allocation. Thus, participants will be asked before all postrandomization interviews with the RA not to reveal anything about their treatment. RAs and therapists will not share the same room, and all therapists involved in the trial will be instructed by the TC not to reveal treatment allocations for participants in any communication with the RAs. The RAs will additionally be asked at each assessment point to guess which treatment was given to each participant, so that the effects of possible bias can be examined in the analysis. Finally, to minimize the risk of bias in relation to treatment knowledge becoming apparent in RAs’ scoring of interview data, RAs’ interviews may be audio-taped, and a random sample may be re-rated by an assessor who has no knowledge of treatment allocation.

First analyses of the trial data will be performed only after all patients have completed the 6-month follow-up. At this point, a data lock will be performed and signed off by the research team and each of the involved mental health care centers, and the locked database will be transferred to an independent trial statistician, as will be attested by (1) the audit trail of the eCRF database and (2) a unique CRC32 hashing code (ie, a checksum algorithm that hashes byte sequences to 32-bit values). All codes will remain unbroken, and all researchers, clinicians, and anyone else involved in the trial will remain blind to treatment outcomes until that time. RAs will remain blind to the treatment condition of patients until all patients have completed the 2-year follow-up.

For the qualitative study, blinding of RAs to treatment condition is not possible, as only patients who have been randomized to the blended condition are interviewed and RAs will also know whether participants have been selected on the basis of how successful they perceived their treatment to have been; it is also highly likely that the latter type of information will be mentioned during the interview. Likewise, RAs will have been in contact with participating therapists, so they will know the therapists and at least to some degree therapists’ opinions and attitudes with regard to blended treatment.

### Statistical Plan and Data Analysis

#### Sample Size and Power Calculation

The primary outcome in this trial is noninferiority of blended care, compared with FTF therapy, in depression as measured by the BDI-II. Sample size and statistical power calculations are based on planned analyses involving the BDI-II. Meta-analytic data suggest a medium ES (Cohen *d/*Hedges *g*=.50) of FTF psychotherapy and blended psychotherapy compared with control conditions, with no differences between FTF or blended PDT and CBT [50,55,97-101]. Our analysis will focus on the difference in effects between blended and FTF treatment and assume that there is no difference between PDT and CBT regarding the effectiveness of either blended or FTF treatment. Thus, the PDT and CBT groups will be combined in the primary analysis.

The tolerance margin for this noninferiority trial was defined to be a maximum of 2 points on the BDI-II. Assuming an SD of 10 points, this is equivalent to a standardized ES of Cohen *d*=0.2, which is considered a minimal meaningful difference in psychosocial interventions [75].

The primary endpoint is the BDI-II score at 6-month follow-up. Noninferiority will be assessed by a mixed-effects analysis of covariance (ANCOVA) that adjusts for therapist variance, baseline BDI-II score, and other baseline covariates. The primary analysis model assumed by the power analysis is:


$$BDI_{ij}=\left(\alpha +u_{j}\right)+\left(\beta \right)BASELINE_{ij}+\;\left(\gamma \right)BLENDED_{ij}+\left(\delta \right)\;x_{ij}^{T}+\;\varepsilon_{ij}$$

where:

- ${BDI}_{ij}$ is the BDI-II score at the primary endpoint of patient *i* treated by therapist *j.*
- *j=1, …, J* indicates the therapists.
- *i=1, …, n_j_* indicates the patients; *n_j_* denotes the number of patients treated by the *j^th^* therapist.
- α is an intercept.
- $u_{j}\sim N(0,\sigma_{u}^{2})$ is a random intercept accounting for differences in outcomes between therapists.
- $\varepsilon_{ij}\sim N(0,\sigma^{2})$ is an error term that accounts for between-patient variability in outcomes.
- ${BASELINE}_{ij}$ is the baseline BDI-II score, which is related to ${BDI}_{ij}$ by slope coefficient β.
- ${BLENDED}_{ij}$ is a dummy variable coded 1=Blended psychotherapy and 0=FTF psychotherapy; the associated coefficient γ estimates the difference in primary outcome between the two groups, adjusted for pretreatment scores and therapist effects.
- $\delta x_{ij}^{T}$ represents further baseline information to be included in the model as controls, where $x_{ij}^{T}$ denotes a column vector of covariate values, and δ denotes a row vector of coefficients.

We will conduct a 1-sided test of noninferiority on the coefficient γ, which represents the difference (after adjustment) between the blended and FTF treatment groups. Formally, our hypotheses will be:

-
$H_{0}:\;\gamma \geq 2$

-
$H_{1}:\;\gamma <2$

The null hypothesis of inferiority will be assessed by a 1-sided *t* test, as is usual for ANCOVA models. The null hypothesis will be rejected if 1-sided *P*<.025, as recommended for noninferiority trials [102].

Power calculations were performed using the standard formula for a 1-sided *t* test for noninferiority trials [103]. The alternative hypothesis used for power calculations was $H_{A}:\gamma =0$, that is, exact equivalence of blended and FTF treatment. The sample size was adjusted to take account of the expected within-patient and within-therapist correlations as follows:


$$n=n^{*}\left(1-r_{within-patient}^{2}\right)\left(1-\;ICC_{therapist}\right)$$

where n* is the sample size required for 80% power without adjustment, and *n* is the actual sample size required in this trial, taking the adjustment into account. Since all other baseline covariates should be approximately equally distributed between randomized groups, their effect on statistical power was assumed to be negligible. Power calculations were confirmed by a user-written simulation in R (R Foundation for Statistical Computing) [104], with 10,000 replications. All simulations agreed with calculations to within 1 percentage point. We made the following assumptions: the within-patient correlation was assumed to be *r_within-patient_*=0.6, which is close to the median value of 0.59 estimated by a meta-analysis of within-patient correlations [105]. We further assumed a therapist effect of *ICC_therapist_*=0.05, which is the current best estimate for therapist effects in trials of psychological treatments [106].

We assumed that 36 therapists would take part in the study. We calculated the smallest sample size needed to achieve at least 80% power, assuming that each therapist treats the same number of patients. This was n*=*504, that is, 14 patients per therapist. This design has 82% power according to our formula (our simulation estimated the power to be 81.6%). Although in reality, the number of patients is unlikely to be exactly the same for each therapist, a simulation study has shown that even far more extreme deviations from equal numbers than are expected in this trial have little effect on the statistical power of mixed-effects models, especially when the intraclass correlation coefficient is small [107].

#### Statistical Analysis Plan

##### Overview

In what follows, the overall data analytic strategy is summarized. A detailed prespecified statistical analysis plan (SAP) for the primary and secondary analyses, as well as moderator and mediator analyses, is provided in Multimedia Appendix 2. This prespecified SAP outlines the analyses concerning primary and secondary outcomes and has not changed substantially compared with the first protocol (version 1.1) approved by the funder on October 25, 2018, that is, before the start of the trial (see also the protocol amendments in Multimedia Appendix 1). These analyses will begin to be performed after all the data from the primary endpoint (6-month follow-up) have been entered, checked, and locked. As outlined earlier, all researchers, clinicians, and anyone else involved in the trial will remain blinded to treatment outcomes until that time. Further analyses focusing on 1-year and 2-year outcomes will be performed after all the data from the 2-year follow-up have been entered, checked, and locked.

##### Primary and Secondary Outcome Analysis

The primary analysis will be an ITT analysis, that is, all participants will be analyzed as randomized, even if they dropped out of treatment or changed treatment during the study period. The primary endpoint is 6 months after the end of treatment. The analysis will focus on the difference between the blended and FTF groups at the primary endpoint:

- Adjusting for patients’ baseline BDI-II score.
- Adjusting for therapy type (CBT or PDT).
- Taking into account between-therapist variation.
- Taking into account variation between treatment centers (as a fixed effect).

A linear mixed-effects model (3-level ANCOVA, with therapists, time points, and patients as levels) will be estimated to investigate the null hypothesis that blended treatment yields inferior outcomes to FTF psychotherapy by 2 points or more on the BDI-II, after adjusting for pretreatment scores and therapist effects. This is equivalent to a standardized ES of 0.2, given the expected baseline SD of 10 for the BDI-II. For details, see the SAP.

This model can accommodate missing values of the outcome variable at either of the 2 time points included in the analysis. Estimates are valid provided that values are missing at random (MAR) conditional on the other variables included in the model. This procedure has been shown to lead to unbiased treatment effect estimates under the MAR assumption and to more efficient estimates compared with multiple imputation [1,108]. The model will be fitted using restricted maximum likelihood estimation.

Analogous to the primary analysis, we will use a mixed-effects logistic regression model to estimate the difference in recovery rates between blended and FTF psychotherapy, and a mixed-effects linear regression model to estimate the difference in participants’ quality of life.

A number of sensitivity analyses will be conducted:

- Using complete cases only. This amounts to estimating Model 1 on participants who provided valid information on the primary outcome at the 6-month follow-up. To be included in this analysis, participants do not need to have provided information on the primary outcome at the end of treatment. Within this sensitivity analysis, item missingness will be handled as described above.
- The assumption of equivalence of effect of blended CBT and PDT will be investigated by estimating a model identical to Model 1 in all respects except that we will allow for an interaction between type of therapy and treatment condition.
  - Whether the conclusions from the primary analysis model (Model 1) would hold if we instead estimated separate treatment effects (blended versus FTF) for CBT and PDT, respectively.
  - Adjusting for concerning imbalances in baseline characteristics.
- Impact of COVID-19. We will document the impact of the COVID-19 pandemic on the delivery of treatments (eg, moving FTF sessions to a remote format), describe the frequency of remote sessions, and conduct exploratory analyses to evaluate whether the difference in effectiveness between the blended and FTF conditions varies depending on whether some FTF sessions were conducted remotely.

Supportive analogous analyses will also be conducted for the 1-year and 2-year follow-up endpoints once they are available after the second database lock, as for the primary endpoint, including all previous endpoints in the linear mixed-effects models.

Furthermore, we will conduct exploratory analyses to determine the shape of the relationship between BDI-II scores and time. On this basis, we will develop a longitudinal mixed-effects model and investigate whether the rate of change in BDI-II differs between (1) blended versus FTF psychotherapy, (2) CBT versus PDT, and (3) the interaction of the two. For the secondary outcomes, we will also explore longitudinal mixed-effects logistic regression to estimate trajectories of recovery rates and longitudinal mixed-effects linear regression to estimate trajectories of quality of life.

##### Moderators and Mechanisms of Change

We will conduct exploratory analyses to investigate potential treatment effect moderators on (1) the primary outcome (BDI-II) at 6-month follow-up, and at 1-year and 2-year follow-up, (2) the secondary outcomes at 6-month follow-up, and at 1-year and 2-year follow-up, and (3) the difference in effectiveness between blended versus FTF treatment, and PDT versus CBT, at 6-month follow-up, and at 1-year and 2-year follow-up. The following moderators will be considered:

- Severity of depression at baseline (assessed by the BDI-II).
- Expectancy of treatment at baseline (assessed by the CEQ).
- Credibility of treatment at baseline (assessed by the CEQ).
- Depressive personality: dependency at baseline (assessed by the DEQ).
- Depressive personality: self-criticism at baseline (assessed by the DEQ).

To investigate these potential moderator effects, we will include all of these potential moderators as predictors in our primary outcome analysis model and assess 95% CIs of the relevant coefficient.

Exploratory analyses will also focus on potential relationships between changes in dependent and self-critical personality features assessed with the DEQ and primary and secondary outcomes, from both a variable-centered and a person-centered perspective.

##### Per-Protocol Analysis

We will repeat the primary analysis as a per-protocol analysis instead of an ITT analysis, using either Model 1 or, if missing outcome values are present, the modification of Model 1 described above. Only participants who have completed the treatment will be included in the per-protocol analysis. Treatment completion is defined as attending at least 8 sessions in the FTF treatments, or attending at least 4 FTF sessions and completing at least 4 modules in the blended treatments.

##### Treatment Fidelity, Feasibility, Acceptability, and Satisfaction

Analyses on treatment fidelity/integrity, feasibility, acceptability, and satisfaction will focus on recruitment rate, retention in treatment, treatment adherence, and adherence to the research protocol, as well as experienced credibility of the treatments and patient satisfaction.

###### Fidelity/Integrity

The integrity of the provision of treatment by therapists will be assessed on the basis of independent ratings of recordings of therapeutic sessions based on the DIT Competence Rating Scale [93] for FTF PDT and an adapted version for blended PDT, and on the Cognitive Therapy Adherence and Competency Scale (CTACS) [109] for FTF CBT. An adapted version of the CTACS will be used for assessing adherence to blended CBT.

###### Feasibility

The feasibility of future studies will be assessed via the proportion of patients who comply with the full clinical and research protocol. Full compliance with the clinical and research protocol is defined as completion of treatment and participation in research interviews at all time points up to the 6-month follow-up. Treatment completion is defined as attending a minimum of 8 sessions in the FTF treatments, or attending at least 4 FTF sessions and completing at least 4 modules in the blended treatments.

###### Acceptability

Acceptability of the treatment to patients will be measured by:

- Treatment retention: the proportion of patients who complete the treatment as defined above.
- Treatment adherence: the proportion of planned sessions attended.

###### Treatment Credibility and Expectancy

Treatment credibility and expectancy will be assessed by using the CEQ [79]. We will document baseline differences (FTF versus blended) in both dimensions and use a multilevel model with random intercepts for sites to test for evidence of systematic differences. We will also compare FTF and blended treatments on credibility and expectancy at the end of treatment and at 6-month follow-up, using regression models analogous to the primary analysis model, including random intercepts for therapists and controlling for baseline differences.

###### Treatment Satisfaction

Satisfaction with treatment will be measured with the CSQ-8 [80]. Again, we will document baseline differences (FTF versus blended) with an adapted version of the CSQ-8 assessing satisfaction with received care before randomization. A multilevel model with random intercepts for sites will be used to test for evidence of systematic differences. We will also compare FTF and blended treatments on treatment satisfaction at the end of treatment and at 6-month follow-up, using regression models analogous to the primary analysis model, including random intercepts for therapists and controlling for baseline differences.

### Qualitative Analyses

Qualitative analyses, as detailed below, will also focus on issues related to treatment acceptability and feasibility, as well as patient satisfaction.

### Interim Analyses

There will be no planned SAE analyses unless there are 5 or more SAEs within a time span of 6 months or any other condition that gives rise to serious concerns regarding the occurrence of SAEs (eg, when there is reason to suspect that the experimental condition may lead to more SAEs). In this event, see the section on SAEs. Adherence to protocol, including the treatment protocol, will be monitored continuously. Any deviation from the planned statistical analyses will be reported in an amendment after it has been discussed and approved by the TSC.

### Health Economic Evaluation

The economic evaluation will be a “piggyback” economic evaluation using outcomes from the trial. Because economic evaluations have a wider time horizon than the trial, and given that beneficial effects can be expected even after 5 years, a Markov model will be used to project future costs and effects using different time horizons, as the higher initial cost of FTF therapy may outweigh the lower cost of blended provision in the long term. The costs of the implementation, costs of usual care, costs in other sectors, patient and family costs, costs of lost production, and judicial costs will be included by using the TIC-P. For the valuation of health care use, standard prices according to published costing guidelines will be used. Medication use and other direct costs will be monitored using national health insurance data provided by RIZIV/INAMI and valued using the listed prices in Belgium. The treatment costs will be estimated using a bottom-up approach. Personnel costs, implementation costs, and overhead costs will be taken into account. In the economic evaluation, outcomes will be expressed in terms of costs per quality-adjusted life year (QALY). Probabilistic analyses that allow multivariate sensitivity analyses will be used [110].

We will also calculate detailed cost-effectiveness plans of the incremental costs in relation to gained QALYs at different willingness-to-pay thresholds, as a basis for future decision-making concerning the implementation of these interventions in mental health care in Belgium.

### Data Collection and Data Analysis in the Qualitative Substudy

#### Data Collection

Semistructured individual interviews with patients will be conducted based on an adaptation of the Experience of Therapy Interview [87], which we have used in similar studies of both FTF and guided internet-based therapy for young people with depression [62,69,87]. Adaptations include questions focusing on patients’ beliefs about the credibility and treatment effects of blended psychotherapy; concerns about privacy and security related to the online modules, their experience with the online modules, the FTF sessions, and the way in which the sessions and modules were integrated; their therapist and the therapeutic relationship; and their experiences with regard to helping and hindering factors associated with blended therapy more generally. As in the original Experience of Therapy Interview [87], the questions are broad, open-ended, and exploratory, to allow participants to tell the story of their experience. The interview guide also includes open-ended follow-up questions to use when elaboration is needed (eg, “Could you tell me a bit more about that?” “Do you remember any specific impasse in therapy where…?”). The interview guide will be piloted with 2 patients to determine whether any further improvements are needed. Focus groups will be conducted using a semistructured interview guide based on an adaptation of the Experience of Therapy Interview [87], based on previous qualitative interview studies of therapists’ experience of providing blended and guided internet-delivered therapy [41,73].

Interviews with patients will be conducted online or in person by a research psychologist with specific training and experience in conducting in-depth, semistructured interviews online. Interviews are expected to take in the range of 1‐1.5 hours. Focus groups with the therapists will be conducted online or in person by a research psychologist with specific training and experience in conducting qualitative research. Focus groups are expected to take in the range of 2‐2.5 hours. All interviews and focus groups will be recorded and transcribed verbatim by an independent RA using Amberscript (Amberscript Global BV), an artificial intelligence-powered software that we have used in previous studies, which is able to automatically transcribe audio- and video-recorded interviews and is fully compliant with General Data Protection Regulation policies.

#### Data Analysis: Thematic Analysis

Data from the qualitative substudy of patients and therapists will be analyzed separately, using TA, a flexible and theory-neutral approach to the analysis of qualitative data. The main aim of TA is to allow the researcher to approach the data with an open mind, and thus to explore and generate rich, context-specific, so-called “thick descriptions” [111] rather than to test specific theories or a priori expectations. Although TA is based on an inductive, bottom-up approach, it is also open to a more top-down, deductive component [112], as in framework analysis [113]. A combined inductive–deductive (or bottom-up and top-down) approach will be adopted in order to remain open to new and potentially unexpected findings, while at the same time exploring the findings from other studies about the subjective experience of blended psychotherapy discussed above.

Consistent with the general approach for the application of TA in psychology [114], a 6-stage approach will be taken for data analysis of the patient interviews. First (stage 1), 2 members of the research team will read all of the transcripts of the interviews to familiarize themselves with the data while taking notes about potentially important findings, themes, or ideas, all the time staying very close to the raw data. Second (stage 2), transcripts of interviews categorized as “helpful” from patients who received blended CBT or blended PDT will be reread by the same 2 members of the research team, and categories that emerge from these data will be coded systematically by both researchers independently; then, transcripts of interviews categorized as “unhelpful” from blended CBT and blended PDT cases will be coded in the same way. After this process, the categories and themes that emerge from these transcripts will be compared between the 2 researchers and discussed. Any differences in interpretation will be resolved through discussion among the 2 researchers and the wider research team. In stage 3, these preliminary qualitative analyses will be discussed by the 2 researchers and collapsed into different subthemes and overarching main themes. Each of these themes will then be labeled and exemplified with quotes from the transcripts (stage 4). This model will then be reviewed by the research team, and any differences in categorizing or labeling will be resolved by consensus, resulting in a final model (stage 5), which will be written up as such (stage 6). A similar data analytic strategy will be followed for the analyses of the focus groups with therapists.

### Ethical Considerations

This trial was approved by the Ethics Committee Research UZ/KU Leuven on January 11, 2019 (approval S59765). This trial was registered on ClinicalTrials.gov (NCT04337242). As mentioned in the Recruitment, Screening, and Gaining Informed Consent section, all patients and therapists will be asked to provide explicit and written informed consent. Personal information will be stored in coded formats only to protect privacy in the study’s eCRF; only the treatment centers will have direct access to uncoded personal information. Access to all coded data will be restricted to authorized study personnel, and strict confidentiality protocols will be followed. Data will be stored in secure, password-protected electronic systems. Participants will receive no compensation for their participation in the study.

### Monitoring and Audit

A full trial monitoring plan was developed and agreed upon by the Trial Management Group and TSC based on the trial risk assessment, which includes regular on-site monitoring. Monitoring is done completely independently from the Trial Management Group and TSC by the UZ Clinical Trials Center (Leuven).

### User Involvement

User involvement is a central component in each stage of the planning and implementation of the trial. Both the trial design and the blended modules were the topic of regular feedback from service users and members of various patient organizations. In addition, the blended modules and online platform used in this study have been the topic of extensive feedback by both therapists and service users since their development. Hence, service users and therapists have been involved at each stage of the adaptation and development of the online modules used in this trial. We will continue to involve service users and therapists during the trial, as we aim to investigate patients’ attitudes and experiences in relation to online mental health care and the combination of online modules and FTF sessions. Even though blended therapy may have major economic benefits, in line with our emphasis on the empowerment of patients, we consider the subjective experience of service users and therapists as central in this trial. A service user representative will also be part of the TSC during the trial.

The 2 overarching themes that emerged from previous rounds of feedback and consultations with service users and patient organizations were the urgent need to increase access to effective treatments for depression in Belgium and to increase the diversity of treatments offered, particularly of psychotherapeutic treatments, as patients felt that choice and the match between patient and type of treatment were very important. This trial heeds this call from service users by evaluating the effectiveness of two different evidence-based treatments for depression (CBT and PDT) offered in two different formats (FTF and blended).

### Management of the Study

The chief investigator will be responsible for the overall management and coordination of the trial across all participating sites and will ensure that the study adheres to the protocol, timelines, and regulatory requirements. A TSC composed of clinical and scientific experts provides strategic guidance and decision-making throughout the study. The committee ensures the integrity and scientific validity of the trial.

## Results

Inclusions started on April 1, 2019, and were seriously delayed because of COVID-19 restrictions as detailed in the protocol amendments (Multimedia Appendix 1). The trial is currently still ongoing; recruitment ended on December 2, 2024. The first data lock (primary outcome) will take place in October 2025, and the results of the primary outcome are expected in February 2026. The second data lock is expected in March 2027, and the results of the 2-year follow-up are expected in September 2027.

## Discussion

### Principal Findings

Depression is a highly prevalent mental health condition. Unfortunately, there still is a considerable treatment gap in that many patients with depression do not seek treatment, delay treatment seeking, or do not receive effective treatment because of limited treatment capacity. Blended psychotherapy, which combines online work and in-person FTF sessions, may help to bridge this treatment gap as a potentially cost-effective treatment that complements other treatments for depression [7,9,10,13-16]. Yet, there is still a paucity of research in this area [9,20-30], particularly concerning the (cost-)effectiveness of blended psychotherapy for depression in patients with more complex presentations [17,19,22,31-37]. Furthermore, little is known about the long-term effects of blended psychotherapy [21,22,31,33], and most research in this area has investigated the effectiveness of blended CBT, with less research on other types of internet-delivered treatments, such as PDT [38]. Finally, more research is needed on moderators and mediators of change and both patients’ and therapists’ experiences of blended treatment.

This multisite trial is expected to provide robust evidence on the effectiveness and cost-effectiveness of blended psychotherapy for depression in adult patients routinely referred to mental health care centers in Flanders, Belgium, compared with traditional FTF psychotherapy. It will also be one of the first trials to directly compare the effectiveness of 2 types of blended psychotherapy (ie, blended CBT and blended PDT) and the effectiveness of these treatments over a clinically meaningful follow-up period of 2 years, which is important given the highly recurrent nature of depression. The trial’s implications may also extend to improving treatment personalization based on moderator and mediator analyses. Secondary analyses, together with the qualitative study, will provide a strong service user perspective on the experience of blended psychotherapy in general and blended psychotherapy for depression in particular. These latter analyses will provide policymakers, researchers, and clinicians with detailed, in-depth information concerning the feasibility, acceptability, and satisfaction associated with FTF and blended psychotherapy for depression, in order to better contextualize and understand findings from this RCT. Taken together, the results will likely inform best practices in psychotherapy delivery and support the development of evidence-based guidelines.

The study’s large sample size and cross-site design promise to enhance the ecological validity of the findings. Other strengths include the study’s rigorous methodology, standardized intervention protocols, and recruitment and treatment delivery in real-world settings. However, potential limitations may include variability in implementation fidelity across sites and challenges in maintaining uniformity in therapist adherence. In addition, results may not generalize to other clinical settings and different health care systems. Despite this, the trial is well positioned to contribute meaningfully to the psychotherapy research landscape and provide a foundation for future studies on scalability and comparative effectiveness.

### Dissemination Plan

To ensure broad impact, the dissemination plan will first focus on the publication of peer-reviewed publications in high-impact clinical and psychological journals to target academic audiences. In addition, presentations will be delivered at national and international conferences. Executive summaries and policy briefs will be shared with health care providers, mental health organizations, and policy stakeholders to inform practice and service delivery. Findings will be communicated to trial sites and participating therapists through tailored webinars and workshops. To reach the general public and patients, we will develop accessible content such as infographics and blog posts shared via institutional websites and social media platforms.

## Supplementary material

10.2196/80511 Multimedia Appendix 1 Protocol amendments.

10.2196/80511 Multimedia Appendix 2 Statistical analysis plan.

10.2196/80511 Checklist 1 SPIRIT checklist.

## Abbreviations

ANCOVA: analysis of covariance
BDI-II: Beck Depression Inventory-II
CBT: cognitive behavioral therapy
CEQ: Credibility and Expectancy Questionnaire
CGG: Centrum Geestelijke Gezondheidszorg
CONSORT: Consolidated Standards of Reporting Trials
CSQ-8: Client Satisfaction Questionnaire-8
CTACS: Cognitive Therapy Adherence and Competency Scale
DEQ: Depressive Experiences Questionnaire
DIT: dynamic interpersonal therapy
*DSM-5*: *Diagnostic and Statistical Manual of Mental Disorders, Fifth Edition*
eCRF: electronic Case Report Form
EQ-5D-5L: EuroQoL 5-Dimension 5-Level
ES: effect size
FTF: face-to-face
ICF: informed consent form
IPAF: interpersonal affective focus
ITT: intention-to-treat
MAR: missing at random
MDD: major depressive disorder
PDT: psychodynamic therapy
PHQ-9: Patient Health Questionnaire-9
QALY: quality-adjusted life year
RA: research assistant
RCT: randomized controlled trial
RIZIV/INAMI: Rijksinstituut voor Ziekte- en Invaliditeitsverzekering/Institut National d’Assurance Maladie-Invalidité
SAE: serious adverse event
SAP: statistical analysis plan
SCID-5-CT: Structured Clinical Interview for DSM-5 disorders – Clinical Trials Version
SF-HLQ: Short Form-Health and Labor Questionnaire
TA: thematic analysis
TC: trial coordinator
TIC-P: Treatment Inventory of Costs in Patients with psychiatric disorders
TSC: Trial Steering Committee
