# Supplementary material for: Clinical and Cost-Effectiveness of Blended Cognitive Behavioral Therapy or Psychodynamic Therapy Versus Face-to-Face Psychotherapy for Depression (BLENDED Study): Protocol for a Pragmatic, Multicenter, Assessor-Blinded Randomized Controlled Noninferiority Trial
Source: JMIR Res Protoc. 2026 Jan 14;15:e80511. doi: 10.2196/80511 (PMC12803439; doi:10.2196/80511)
Supplement: Multimedia Appendix 1 [file resprot-v15-e80511-s001.docx]

The following amendments were made to the original trial protocol (Version 1.1, dated 25 October 2018):

- A first amendment clarified the visit windows for client follow-ups in the protocol, specifying that assessments could take place ±2 months either side of the ideal follow-up time point. In addition, data protection measures in the ICF were further specified for patients. The EC Research UZ/KU Leuven approved these changes to the original protocol (Version 1.1) and the ICF on 27 February 2019.
- A second set of amendments clarified two exclusion criteria, as well as the frequency of therapy sessions. One exclusion criterion concerned the use of antipsychotic medication. As this type of medication is often used to treat depressive symptoms in patients without primary psychotic symptoms, the use of antipsychotic medication was allowed, but not if it was prescribed for primary psychotic symptoms. The second exclusion criterion concerned the presence of suicidal thoughts and attempts in the past or present. As suicidal thoughts and self-injurious thoughts and behaviors are extremely common in depression, this exclusion criterion was further specified as: “Historic or current self-injury/parasuicide of such extent and/or severity that may substantially interfere with the ability to engage in brief psychotherapy”. Finally, the original protocol specified that the frequency of FTF therapy sessions was weekly in the FTF condition and every 2 weeks in the blended condition. However, as it was not uncommon for sessions to deviate from the theoretical weekly or 2-weekly frequency because of annual leave and sick leave of therapists and/or patients, it was specified that, through mutual agreement between patient and therapist, session frequency could deviate from the weekly/2-weekly schedule. The EC Research UZ/KU Leuven approved the above changes (protocol Version 1.2, dated 15/12/2019) on 20 January 2020.
- As a result of the onset of the COVID-19 pandemic, we were forced to make a number of changes to the protocol and inform patients of these changes through an addendum to the ICF. During the lockdown in Belgium from mid-March to mid-June 2020, physically attending screening and therapeutic sessions was not possible and/or advised against, so for many patients FTF sessions were replaced by remote consultation (by phone or online) for screening and psychotherapy. These remote FTF sessions were considered equivalent to in-person/on-site FTF sessions. In addition, some potentially eligible patients and patients already in the study were unable or unwilling to make the switch to remote consultations, opting to wait until on-site visits and FTF sessions were allowed again. End-of-treatment visits (and subsequent follow-up assessments) that would fall outside the predefined time window for end-of-treatment visits (ie, 6 ± 2 months) were postponed to correspond to the exact period of COVID-19-related delay (in weeks; documented in eCRF). Finally, as part of the measures implemented to reduce the impact of the COVID-19 pandemic, before screening, patients were initially offered the option to digitally sign the ICF or, if digital signing was impossible for patients, they could confirm their informed consent by email. In these cases, physical informed consent signatures were collected at the first possible visit to the mental health center. The EC Research UZ/KU Leuven approved these changes (protocol Version 1.3, dated 16 October 2020) on 11 May 2021.
- Finally, additional funding for a small qualitative study, focusing on the subjective experience of patients in the blended care condition in the BLENDED trial, was obtained. Additions were made to the protocol to include this qualitative sub-study in the protocol and an additional addendum to the ICF was made for patients. These amendments and the protocol version (Version 1.4, dated 15 November 2024) were approved by the EC Research UZ/KU Leuven on 12 December 2024.

None of these amendments is substantive, with the possible exception of the COVID-19 pandemic-related measures and particularly the move to remote sessions, which will be addressed by sensitivity analyses as detailed in the SAP. None of these changes was made based on the trial results as they were made and approved by the EC before the data lock, as can also be demonstrated based on version control with protocol identifiers and dates.
