# Supplementary material for: Clinical and Cost-Effectiveness of Blended Cognitive Behavioral Therapy or Psychodynamic Therapy Versus Face-to-Face Psychotherapy for Depression (BLENDED Study): Protocol for a Pragmatic, Multicenter, Assessor-Blinded Randomized Controlled Noninferiority Trial
Source: JMIR Res Protoc. 2026 Jan 14;15:e80511. doi: 10.2196/80511 (PMC12803439; doi:10.2196/80511)
Supplement: Multimedia Appendix 2 [file resprot-v15-e80511-s002.docx]

Blended Care vs. Face-to-Face Therapy for Depression (BLENDED):

Statistical analysis plan

Peter Martin and the BLENDED team

Version history log

| Version | Date | Details of Change |
| --- | --- | --- |
| 01 | 10 March 2025 | - |
|  |  |  |

# Study Summary

| For full details see the trial protocol (BLENDED study protocol Version 1.4, from 15 Nov 2024) | |
| --- | --- |
| **Title** | Blended Care Psychodynamic Therapy or Cognitive Behavioral Therapy versus Face-to-Face Psychotherapy for Depression: A pragmatic multicenter randomized controlled non-inferiority trial |
| **Short title** | Blended Care vs Face-to-face Therapy for Depression (BLENDED) |
| **Chief Investigators** | Prof. Patrick Luyten |
| **Statistician** | Dr Peter Martin |
| **Design** | An individually randomised controlled trial with adults receiving psychotherapy for depression. Individuals are randomly allocated to therapists, who are qualified either to provide cognitive behavioural therapy or psychodynamic therapy, and then randomly allocated to either Blended Care or Face-to-face psychotherapy. |
| **Primary objective** | To evaluate whether Blended Care is non-inferior to Face-to-face psychotherapy in the treatment of adult depression. |
| **Population** | Adults attending Centres for Health Care (‘Centra Geestelijke  Gezondheidszorg’, CGGs) in Flanders, Belgium. Target sample size: 504. |
| **Study Type** | Interventional randomised controlled trial |
| **ClinicalTrials.gov** | Registration number: NCT04337242 |

|  |  |
| --- | --- |

# Introduction

## Purpose and scope of the statistical analysis plan

This Statistical Analysis Plan was written by Peter Martin and follows the guidelines outlined in Gamble et al. (2017). This document describes the main statistical analyses to be applied to the data from the BLENDED trial, but does not cover health economic analyses or the qualitative sub-study embedded within the BLENDED trial. Further statistical analyses mentioned in the trial protocol, such as those relating to the investigation of treatment mechanisms, are also not covered in this analysis plan.

## Timing of analysis

The analyses described within this analysis plan will begin to be performed after all the data from the primary endpoint (6-months follow-up) have been entered, checked and locked and this analysis plan has been finalised. Further analyses will be performed after all the data from the 2-year-follow up have been entered, checked and locked.

## Data checking

Before analysis and database lock, basic checks will be performed on the quality of the data, focusing on identifying:

- Missing data
- Data outside expected range
- Other inconsistencies between variables, e.g. in the dates the questionnaires were completed

If any inconsistencies are found, the corresponding values will be double checked with the researchers and corrected if necessary in the source data. This checking process and subsequent changes will be documented.

# Description of the trial

## 3.1. Intervention

BLENDED is a non-inferiority trial investigating the hypothesis that blended psychotherapy, which combines face-to-face psychotherapy with online support, is non-inferior to classic face-to-face (F2F) psychotherapy. (See the trial protocol for details.) Therapists from two therapy types take part in the trial: Psychodynamic Therapy (PDT) and Cognitive Behavioural Therapy (CBT). Each therapist provides either blended or face-to-face treatment, according to which treatment each patient is randomized to. Contamination is thought unlikely as face-to-face therapeutic technique does not vary between the conditions, and since patients in the F2F condition do not have access to the online support materials that characterize the blended condition. Therapists are either PDT or CBT practitioners, so each therapist provides the same therapy type to all their patients.

## 3.2. Randomization

This trial will employ stratified randomization of participants to one of four conditions: Blended CBT, Blended PDT, F2F CBT, or F2F PDT. Randomization will be carried out in two stages and allow for stratification by site. At stage 1, patients will be randomized to therapists within each Centre for Health Care (*Centrum Geestelijke Gezondheidszorg*, CGG), using permuted blocks. As therapists are only providing either CBT or PDT, stage 1 implicitly allocates patients to therapy type. At stage 2, patients will be randomized to either FTF or blended care, again using permuted blocks. The assumptions of this pragmatic study are that PDT and CBT are equally effective, and that any difference in the effect of blended versus F2F treatment is the same in both PDT and CBT. These assumptions will underpin the primary analysis, but sensitivity analyses will be conducted to investigate whether the assumptions hold and if the primary conclusions would change if, for example, the treatment effect difference (blended versus F2F) was not the same in PDT and CBT.

##

## 3.3. Duration of the treatment period and frequency of follow-up

Outcome measures will be collected at the following time points:

- T1: Baseline (prior to randomization)
- T2: End of treatment
- T3: 6 months after treatment end (**primary endpoint**)
- T4: 12 months after treatment end
- T5: 24 months after treatment end

“Visit windows” of ± 2 months either side of the ideal follow-up time point are specified in the trial protocol.

# Data collection

## 4.1. Participant characteristics

The following demographic characteristics will be collected at baseline: age, sex, level of education, civil status, employment, nationality (defined as Belgian or non-Belgian). Baseline data will also include information about previous health service use, including pharmacotherapy, attitudes towards therapy (CEQ), and satisfaction with care prior to treatment start (adapted CSQ-8).

## 4.2. Outcome data

### 4.2.1 Primary outcomes

The primary outcome of the study is the Beck Depression Inventory (BDI-II). The primary endpoint is 6 months after end of therapy (T3).

### 4.2.2 Secondary outcomes

The secondary outcomes are recovery from depression and health-related quality of life. There are three secondary outcome measures:

- Recovery from depression:
  - Structured Clinical Interview for DSM-V, Clinical Trials Version (SCID-5-CT)
  - Patient Health Questionnaire-9 (PHQ-9)
- Health-related quality of life
  - EQ-5D-5L

The SCID-5-CT gives a binary variable indicating whether a participant meets diagnostic criteria for major depression. PHQ-9 gives a score ranging from 0 to 27. A cut-off of 10 or higher will be used to indicate ‘major depression’ (non-recovery; Levis et al., 2019).

The EQ-5D-5L score will be calculated from the participants’ responses using the value set for Flanders (Cleemput, 2010) developed for the EQ-5D-3L, which will be converted to the EQ-5D-5L using the method of van Hout (2012).

## 4.3. Fidelity

Integrity of the treatment provision by therapists will be assessed on the basis of independent ratings of recordings of therapeutic sessions based on the DIT Competence Rating Scale (Lemma et al., 2024) for both F2F and blended PDT, and on the Cognitive Therapy Adherence and Competency Scale (CTAC, Barber et al., 2003) for F2F CBT. An adapted version of the CTAC will be used for blended CBT.

## 4.4. Feasibility

Feasibility of future studies will be assessed via the proportion of patients who comply with the full clinical and research protocol. Full compliance with the clinical and research protocol is defined as completion of treatment and participating in research interviews at all time points up to the 6-months follow-up. Treatment completion is defined as attending a minimum of 8 sessions in the F2F treatments and attending at least 4 F2F sessions and completing at least 4 modules in the blended treatments.

## 4.5. Acceptability

Acceptability of the treatment to patients will be measured by

- Recruitment rate
- Treatment retention: the proportion of patients who completed the treatment (defined as above under point 4.4);
- Treatment adherence: the proportion of planned sessions attended.

## 4.6. Treatment credibility and expectancy

Treatment credibility and expectancy will be assessed by the Credibility and Expectancy Questionnaire (CEQ; Devilly & Borkovec, 2000). This yields two sub-scales: credibility and expectancy. We will document baseline differences (F2F vs Blended) in both dimensions and use a multilevel model with random intercepts for sites to test for evidence of systematic differences. We will also compare F2F and blended treatments on credibility and expectancy at the end of treatment and at 6-months follow-up, using regression models analogous to the primary analysis model (see below, section 5.5), including random intercepts for therapists and controlling for baseline differences.

## 4.7. Treatment satisfaction

Satisfaction with treatment will be measured with the Client Satisfaction Questionnaire-8 (CSQ-8; Attkisson & Greenfield, 2004). We will document baseline differences (F2F vs Blended) with an adapted version of the CSQ assessing satisfaction with received care before randomization. We will use a multilevel model with random intercepts for sites to test for evidence of systematic differences. We will also compare F2F and blended treatments on treatment satisfaction at the end of treatment and at 6-months follow-up, using regression models analogous to the primary analysis model, including random intercepts for therapists and controlling for baseline differences.

# Data analysis

Analyses will be carried out based on the intention to treat (ITT) principle, comparing the groups as randomised regardless of compliance with the intervention. The primary analysis will be performed on observed outcome values (without imputation, except missing item imputation discussed below to enable us to calculate total scores). Confidence intervals will be symmetric around the point estimate, using the 95 % level of confidence.

## 5.1. Recruitment and representativeness of recruited patients

A consort diagram will be constructed to describe the flow of subjects through the trial (http://www.consort-statement.org/). The diagram will detail the number of subjects: invited to participate; agreeing to enter the study (with reasons for refusal); receiving the intervention (with reason for not receiving this); followed up and withdrawn (with reasons).

## 5.2. Baseline characteristics

Baseline characteristics of the participants will be summarised by treatment group to gauge the balance in characteristics between the randomised groups. The results will be presented as means, standard deviations, medians and inter-quartile ranges for numeric variables; and frequencies and percentages for categorical variables. No statistical hypothesis testing will be used.

## 5.3. Adherence to treatment, attrition and missing data

**Adherence**. Adherence will be measured as the proportion of treatment sessions attended. The mean, range, and interquartile range of this proportion will be reported.

**Attrition**. Some loss to follow-up is expected over twelve months. Reasons for missing outcome data will be described and frequency (%) of subjects with missing data, by reason will be provided for each randomised group (and for each outcome).

## 5.4. Adverse event reporting

Procedures for monitoring adverse events are outlined in the trial protocol.

## 5.5. Analysis of primary outcomes

Baseline, post-intervention, and 6-month follow-up scores on the BDI-II will be summarized separately for each of the four treatment groups (CBT Blended, PDT Blended, CBT F2F, PDT F2F) using means, standard deviations, and quartiles.

The primary endpoint is 6 months after end of therapy (T3). The analysis will focus on the difference between the BLENDED and F2F groups at the primary endpoint,

- adjusting for patient’s baseline BDI-II score,
- adjusting for therapy type (CBT or PDT),
- taking into account between-therapist variation, and
- taking into account variation between treatment centres (as a fixed effect).

The primary analysis will be an intention to treat (ITT) analysis. That is, all participants will be analysed as randomized, even if they dropped out of treatment or changed treatment during the study period.

The model for the primary analysis is a linear mixed effects model (3-level analysis of covariance). The model is:

$${BDI}_{ijk}=\left( \beta_{0}+u_{jk}+u_{k} \right)+ \left( \beta_{1} \right){BLENDED}_{jk}+\left( \beta_{2} \right){TIME}_{ijk}+ \left( \beta_{3} \right){BLENDED}_{jk}\times{TIME}_{ijk}+ \left( \beta_{4} \right){BDI.0}_{jk}+\left( \beta_{5} \right){CBT}_{j}+\left( \beta_{6} \right){CBT}_{j}\times{TIME}_{ijk}+\sum_{m=1}^{M} \left( \gamma_{m} \right)X_{m,ijk}+ \varepsilon_{ijk}$$

**(Model 1)**

where

- ${BDI}_{ijk}$ are the BDI-II scores at the primary endpoint; a higher score indicates the presence of more severe symptoms of depression;
- *k = 1, …, K* indicates the therapists*;*
- *j = 1, …, n_k_* indicates the patients; *n_k_* denotes the number of patients treated by the *k^th^* therapist;
- *i =* 2, 3 indicates the time points; 2 denotes T2 (end of treatment), 3 denotes T3 (primary end point);
- $\beta_{0}$ is the fixed intercept;
- $u_{jk}\sim N(0, \sigma_{u.jk}^{2})$ is a random intercept accounting for differences in outcomes between patients;
- $u_{k}\sim N(0, \sigma_{u.k}^{2})$ is a random intercept accounting for differences in outcomes between therapists;
- $\varepsilon_{ij} \sim N(0, \sigma^{2})$ is a random error that accounts for between-patient variability in outcomes;
- ${BLENDED}_{jk}$ is the treatment indicator variable, coded 1 for blended psychotherapy and 0 for face-to-face psychotherapy; the associated coefficient $\beta_{1}$ represents the difference in primary outcome between the two groups at the primary endpoint;
- ${TIME}_{ijk}$ is the time indicator, coded 0 for the primary endpoint, and 1 for end of treatment;
- ${BLENDED}_{jk}\times{TIME}_{ijk}$ specifies the interaction of treatment and time; the associated coefficient represents the difference in treatment effectiveness between the two time points;
- ${BDI.0}_{jk}$ is the baseline BDI-II score;
- ${CBT}_{j}$ is the therapy type, coded 1 for CBT and 0 for PDT (this variable does not have an index *k* since it does not vary within therapist); ${CBT}_{j}\times{TIME}_{ijk}$ specifies the interaction of therapy type by time;
- $X_{m,ijk}$ are dummy variables identifying sites (regions) with associated slope coefficients $\gamma_{m}$, numbered *m* = 1, …, 8 (as there are nine sites).

This model can accommodate missing values of the outcome variable at either of the two time points included in the analysis. Estimates are valid provided that values are missing at random (MAR) conditional on the other variables included in the model. This procedure has been shown to lead to unbiased treatment effect estimates under the MAR assumption, and to more efficient estimates compared to multiple imputation (Sullivan et al., 2018: Section 5). The model will be fitted using Restricted Maximum Likelihood (REML) estimation.

The primary analysis will investigate the null hypothesis that blended care yields inferior outcomes to face-to-face psychotherapy by 2 points or more on the BDI-II, after adjusting for pre-treatment scores, therapy type, therapist and site effects. This is equivalent to a standardised effect size of 0.2, given the expected baseline SD of 10 for the BDI-II. We will conduct a one-sided test of non-inferiority on the coefficient $\beta_{1}$. Formally, we will test: $H_{0}: \beta_{1}\geq2$ vs $H_{1}: \beta_{1}<2$.

The null hypothesis of inferiority will be rejected if *p_one-sided_ < 0.025*, as recommended for non-inferiority trials (Leichsenring et al., 2015; Wellek, 2010). We will also calculate a 95 % confidence interval for$\beta_{1}$. In addition, we will calculate the estimated standardized effect size (Blended vs F2F), based on the pre-treatment standard deviation, with 95 % confidence interval.

## 5.6. Analysis of secondary outcomes

Analogous to the primary analysis, we will use a mixed effects logistic regression model to estimate the difference in recovery rates between Blended and F2F psychotherapy, and a mixed effects linear regression model to estimate the difference in quality of life. Since patients by default meet criteria for depression at T1, baseline diagnostic status won’t need be taken into account in the analyses of recovery. Baseline EQ-5D-5L measurements will be taken into account. Rather than performing a non-inferiority test, interpretation will focus on the 95 % confidence intervals for the ratio of the odds of recovery in Blended vs F2F therapy on PHQ-9 and SCID, and the 95 % confidence interval for the difference, Blended vs F2F, in health-related quality of life (EQ-5D-5L).

The results for the secondary outcomes will be presented as estimates with 95% confidence intervals. Analyses will compare groups defined by intention to treat and include all those with available data. Analyses for post-intervention outcomes will be conducted alongside the primary analysis. Analyses for twelve-month and two-year follow-up data will be conducted when data will have become available.

## 5.7. Missing items in scales and subscales of primary and secondary outcomes

Missing items within summary scales (e.g. the BDI-II) will be prorated if item missingness is < 2 % in the sample as a whole, and fewer than 10 % of individuals have more than 1 item missing. If either total item missingness ≥ 2 % or at least 10 % of individuals have more than one item missing, we will conduct multiple imputation of missing values.

## 5.8. Sensitivity and other planned analyses

### 5.8.1 Sensitivity analyses

**Complete cases**

A sensitivity analysis using complete cases approach will be conducted. This amounts to estimating Model 1 on those participants who provided valid information on the primary outcome at the 6-months follow-up (T3). To be included in this analysis, participants do not need to have provided information on the primary outcome at T2. Within this sensitivity analysis, item missingness will be handled as above.

**Assumption of equivalence of effect of Blended Care in CBT and PDT**

Model 1, as outlined above, assumes that CBT and PDT are equivalent with respect to the difference in effectiveness of blended care versus face-to-face therapy. To test this assumption, we will estimate a model identical to model 1 in all respects, except that we will allow for an interaction between type of therapy and treatment condition, as follows:

${BDI}_{ijk}=\{Model 1\}+\left( \beta_{7} \right){CBT}_{j}\times{BLENDED}_{jk}+\left( \beta_{8} \right){CBT}_{j}\times{BLENDED}_{jk}\times{TIME}_{ijk}$ **(Model 2)**

Model 2 allows for a difference between CBT and PDT with respect to relative effectiveness of blended and face-to-face psychotherapy, at each of T2 and T3. We will carry out an equivalence test of the coefficient $\beta_{7}$ , testing the null hypothesis: $\beta_{7}\leq-2$ or $\beta_{7}\geq2$, vs the alternative hypothesis: $-2<\beta_{7}<2$. Rejection of this null hypothesis would be interpreted as evidence for the equivalence of the effect of Blended Treatment in CBT and PDT. We will also calculate a 95 % confidence interval for the coefficients $\beta_{7}$ and $\beta_{8}$to gauge their likely size.

We will conduct a sensitivity analysis to gauge whether the conclusions from the primary analysis model, Model 1, would hold if we instead estimated separate treatment effects (blended vs F2F) for CBT and PDT, respectively. Let $\beta_{1,M2}$ denote the coefficient of the indicator variable “Blended” in Model 2. This coefficient represents the difference in the effect of Blended vs F2F treatment for patients receiving PDT. The non-inferiority test on coefficient $\beta_{1, M2}$ will be conducted to investigate whether our conclusions would change for PDT had we adopted Model 2. By changing the reference category of our therapy type indicator variable (such that CBT = 0 and PDT = 1), we can perform the analogous test on the effect of Blended vs FTF treatment in CBT, calling this re-parameterized model “Model 2*”, and the treatment condition coefficient in this model, $\beta_{1, M2}^{*}$. We will also calculate 95 % confidence intervals for $\beta_{1,M2}$ and $\beta_{1, M2}^{*}$.

**Adjusting for baseline characteristics**

For the primary and secondary outcomes using the same modelling approaches as described previously, the treatment effect will be estimated adjusting for any concerning imbalances in baseline characteristics.

**Impact of Covid-19 pandemic**

The Covid-19 pandemic, and associated public health measures such as advice against in-person meetings, occurred during the treatment period for many participants in this trial. This led to therapy sessions being cancelled and also to F2F therapy sessions being conducted online, rather than in-person. We will document these cases, describe their frequency, and conduct exploratory analyses to evaluate whether the difference in effectiveness between Blended and F2F conditions varies depending on whether some F2F sessions were conducted online.

### 5.8.2 Supportive analyses

**Other endpoints**

We will perform analogous analyses, as for the primary endpoint, for the secondary endpoint “end of treatment” (T2), using the estimates from Model 1.

Once data for endpoints T4 and T5 (12-months and 24-months follow-up) are available, we will conduct analogous analyses as for the primary endpoint, including all previous endpoints in the linear mixed effects models.

**Longitudinal analyses**

Furthermore, we will conduct exploratory analyses to determine the shape of the relationship between BDI-II score and time. On this basis we will develop a longitudinal mixed effects model and investigate whether the rate of change in BDI-II scores differs between

- Blended vs F2F psychotherapy,
- CBT vs PDT, and
- the interaction of the two.

The interaction allows us to investigate the difference between Blended and F2F psychotherapy separately for CBT and PDT. Rather than performing inferiority tests, the analyses in this model will focus on estimating the sizes of the parameters of interest, using 95 % confidence intervals.

For the secondary outcomes, we will also explore longitudinal mixed effects logistic regression to estimate trajectories of recovery rates and longitudinal mixed effects linear regression to estimate trajectories of quality of life.

**Treatment effect moderators**

We will conduct exploratory analyses to investigate the following potential treatment effect moderators:

- Severity of depression at baseline (assessed by the BDI-II)
- Expectancy of treatment at baseline (assessed by the CEQ)
- Credibility of treatment at baseline (assessed by the CEQ)
- Depressive personality: dependency at baseline (assessed by the DEQ) (Fichman et al., 1994)
- Depressive personality: self-criticism at baseline (assessed by the DEQ)

We will investigate whether these pre-treatment patient characteristics are related to

1. the primary outcome (BDI-II) at 6-months follow-up,
2. the secondary outcomes at 6-months follow-up,
3. the difference in primary and secondary outcomes between blended vs F2F, and PDT vs CBT at 6-months follow-up.
4. the rate of change in the primary and secondary outcomes.

To investigate questions (i) and (ii), we will include all of these potential moderators as predictors in our primary outcome analysis model, and assess 95 % confidence intervals of the relevant coefficient. Cut-off points to discretise the interval-level moderators will be explored. To investigate question (iii), we will add an interaction between the potential moderator and the treatment indicator variable, separately for each potential moderator, and assess 95 % confidence intervals of the coefficient of the interaction. These analyses will clearly be labelled as exploratory. Once data from the 12-months and 24-months follow-ups are available, these analyses will be extended to include those additional data.

**Per protocol analysis**

We will repeat the primary analysis as a per protocol analysis, instead of an ITT analysis, using either Model 1 or, if missing outcome values are present, the modification of Model 1 described above in the section on missing values. Only participants who have completed the treatment they were randomized to will be included in the per-protocol analysis. Treatment completion is defined as attending at least 8 sessions in the F2F treatments and attending at least 4 F2F sessions and completing at least 4 modules in the blended treatments.

## 5.9. Model checking

The linear mixed effects models assume that the residuals are normally distributed and homoscedastic. This will be checked using residual plots. Since our outcome measures are scales with fixed minima and maxima, outliers with high leverage are unlikely to occur, but we will check for influential observations also. If substantial departures from normality or homoscedasticity occur, additional sensitivity analyses using suitable transformations of the relevant outcome variables will be considered.

## 5.10. Convergence issues

If the REML estimation for the three-level outcome model does not converge, we will instead estimate a two-level model (patients within therapist) using primary endpoint (T3) measurements only. T2 measurements won’t then be used in the model, and the variable ‘time’ and the associated interactions will be dropped. However, missing T3 measurements will be imputed using all variables included in the model as well as T2 measurements. If this model also fails to converge, we will fit a single-level model without therapist effects.

# References

80. Attkisson, C. C., & Greenfield, T. K. (2004). The UCSF Client Satisfaction Scales: I. The Client Satisfaction Questionnaire-8. In M. E. Maruish (Ed.), *The use of psychological testing for treatment planning and outcomes assessment: Instruments for adults* (3rd ed., pp. 799–811). Lawrence Erlbaum.

109. Barber, J. P., Liese, B. S., & Abrams, M. J. (2003). Development of the cognitive therapy adherence and competence scale. *Psychotherapy Research*, *13*(2), 205–221. https://doi.org/10.1093/ptr/kpg019

115. Cleemput, I. (2010). A social preference valuations set for EQ-5D health states in Flanders, Belgium. *European Journal of Health Economics*, *11*(2), 205–213. https://doi.org/10.1007/s10198-009-0167-0

79. Devilly, G. J., & Borkovec, T. D. (2000). Psychometric properties of the credibility/ expectancy questionnaire. In *Journal of Behavior Therapy and Experimental Psychiatry* (Vol. 31).

84. Fichman, L., Koestner, R., & Zuroff, D. C. (1994). Depressive Relation to Trends Styles in Adolescence: Assessment, Social Functioning, and Developmental. In *Journal of Youth and Adolescence* (Vol. 23, Issue 3).

116. Gamble, C., Krishan, A., Stocken, D., Lewis, S., Juszczak, E., Doré, C., Williamson, P. R., Altman, D. G., Montgomery, A., Lim, P., Berlin, J., Senn, S., Day, S., Barbachano, Y., & Loder, E. (2017). Guidelines for the content of statistical analysis plans in clinical trials. *JAMA - Journal of the American Medical Association*, *318*(23), 2337–2343. https://doi.org/10.1001/jama.2017.18556

102. Leichsenring, F., Luyten, P., Hilsenroth, M. J., Abbass, A., Barber, J. P., Keefe, J. R., Leweke, F., Rabung, S., & Steinert, C. (2015). Psychodynamic therapy meets evidence-based medicine: A systematic review using updated criteria. *The Lancet Psychiatry*, *2*(7), 648–660. https://doi.org/10.1016/S2215-0366(15)00155-8

93. Lemma, A., Hepworth, M., Fonagy, P., Abrahams, D., & Luyten, P. (2024). *Brief dynamic interpersonal therapy: A clinician’s guide* (2nd ed.). Oxford University Press.

117. Levis, B., Benedetti, A., & Thombs, B. D. (2019). Accuracy of Patient Health Questionnaire-9 (PHQ-9) for screening to detect major depression: Individual participant data meta-analysis. *The BMJ*, *365*. https://doi.org/10.1136/bmj.l1476

108. Sullivan, T. R., White, I. R., Salter, A. B., Ryan, P., & Lee, K. J. (2018). Should multiple imputation be the method of choice for handling missing data in randomized trials? *Statistical Methods in Medical Research*, *27*(9), 2610–2626. https://doi.org/10.1177/0962280216683570

118. Van Hout, B., Janssen, M. F., Feng, Y. S., Kohlmann, T., Busschbach, J., Golicki, D., Lloyd, A., Scalone, L., Kind, P., & Pickard, A. S. (2012). Interim scoring for the EQ-5D-5L: Mapping the EQ-5D-5L to EQ-5D-3L value sets. *Value in Health*, *15*(5), 708–715. https://doi.org/10.1016/j.jval.2012.02.008

103. Wellek, S. (2010). *Testing statistical hypotheses of equivalence and noninferiority* (2nd ed.). CRC Press.
